# Personal Protective Equipment Immediately Alters the Core–Skin Temperature Gradient in Recruit Firefighters

**DOI:** 10.3390/life16040671

**Published:** 2026-04-14

**Authors:** William R. Kinnaird, Andrew R. Moore, Tiffany J. Oberther, A. Maleah Winkler

**Affiliations:** Department of Kinesiology, Augusta University, 3109 Wrightsboro Road, Augusta, GA 30909, USA; wkinnaird@augusta.edu (W.R.K.); andmoore@augusta.edu (A.R.M.); tiffjune@outlook.com (T.J.O.)

**Keywords:** tactical athletes, thermoregulation, heat stress, PPE, temperature monitoring, occupational stress

## Abstract

The purpose of this study was to quantify the degree to which personal protective equipment (PPE) affects the core–skin temperature gradient in nine recruit firefighters attending fire academy. Participants wore a chest monitor and ingested a pill to continually measure skin and core temperature, respectively. PPE status was defined as periods during which participants were wearing full PPE (ON) and not wearing PPE (OFF). During the study, participants transitioned between PPE ON to PPE OFF seven times. These transition timepoints, defined as paired two-minute averages collected immediately before (PPE OFF) and shortly after (PPE ON) donning PPE, were analyzed to examine the immediate effects of PPE use. Factorial repeated-measures ANOVAs were used to assess the effects of PPE status, time, and their interaction on temperature gradient, core temperature, and skin temperature. There was a significant interaction effect on the temperature gradient (*p* < 0.001), with higher gradients in the OFF condition compared to ON at timepoints 2–7. A significant interaction was observed for skin temperature with higher values in ON than OFF for timepoints 2–7 as well. There was no interaction (*p* = 0.445) or main effect of PPE status (*p* = 0.906) for core temperature. This study demonstrates that adding PPE significantly reduced the core–skin temperature gradient in recruit firefighters, largely due to increases in skin temperature.

## 1. Introduction

Firefighting is a dangerous occupation that requires individuals to perform demanding physical labor in extreme and hazardous conditions. The hazards of firefighting include physical injuries (cuts, falls, collisions), toxic exposure, chaotic work environments that often include sleep restriction and poor dietary intake, and thermal dangers such as flame and extreme heat exposure [1]. While these hazards tax the whole body, the cardiovascular system appears to be the most affected [2]. Once on scene, firefighters perform physically demanding work, often resulting in near-maximal heart rates (i.e., 85–100% of their age-predicted maximum) and elevated blood pressures for prolonged periods of time (i.e., 20–40 min during active fire suppression), while being exposed to extreme temperatures that can range from 50 to 100 °C [2]. Additionally, studies have shown that heart rate stays elevated during short typical rest intervals and after leaving the fireground which may indicate sustained cardiovascular strain [3]. Together, this combination of cardiovascular strain and environmental heat stress places exceptional demands on the thermoregulatory system, increasing fatigue and the risk of heat-related illness.

Under normal conditions, the body tightly regulates core temperature between approximately 35.7 °C and 37.3 °C to ensure proper physiological function [4]. Core temperature reflects the temperature of the internal organs and is maintained via homeostatic control through the integration of core and peripheral thermoreceptors within the central nervous system [5]. Because core temperature reflects the body’s ability to maintain homeostasis, it serves as the primary indicator of internal heat strain and the body’s ability to tolerate metabolic and environmental heat loads; core temperature measures are therefore the basis for heat illness classifications and safety guidelines [5,6].

Human heat production is primarily determined by metabolic activity that includes basal metabolic rate, muscular work, and cellular chemical reactions, which increase with increases in cellular temperature [7]. During physical work, metabolic heat production substantially increases, which leads to proportionate increases in core temperature relative to the intensity of work being done [7,8]. To maintain homeostatic core temperatures the body must offload this internalized heat [4,7,9]. Cutaneous vasodilation allows for warm blood to travel from core to skin where it can then increase sweat production and ideally evaporation. Unlike core temperature, skin temperature is strongly influenced by environmental factors like humidity, airflow, and ambient temperature [8,10,11]. When ambient temperature exceeds skin temperature, the body gains heat via conduction and radiation, making evaporation the primary method for heat dissipation [7]. Thus, while core temperature is primarily dependent on metabolic work, skin temperature typically adjusts in response to environmental conditions. The balance that is the core–skin temperature gradient is crucial for ensuring efficient heat transfer from the body to the environment.

In firefighting, these thermoregulatory processes are impaired by the use of personal protective equipment (PPE) [12]. The National Fire Protection Association requires the use of PPE consisting of a protective jacket, pants, gloves, boots, helmet, and a self-contained breathing apparatus to protect firefighters from the many hazards associated with firefighting [13]. PPE creates a hot, humid microenvironment with minimal airflow that severely limits evaporative and convective heat loss. This restriction promotes the storage of metabolic heat leading to progressive increases in both skin and core temperatures during work [7]. These thermoregulatory demands are compounded by the physical demands of the gear itself. PPE has been shown to impair anaerobic capacity, with meaningful reductions to both sprint performance and explosive power [14]. Aerobically, PPE substantially decreases exercise tolerance while also increasing the metabolic costs of movement by adding mass and changing movement patterns [15,16]. Consequently, firefighters experience consistently higher heart rates and greater cardiovascular strain when performing tasks in PPE compared to no PPE [9]. Firefighting tasks such as search and rescue, stair climbing, and hose advancement, when performed in full PPE and self-contained breathing apparatus, can require energy expenditures equivalent to 80–100% of a firefighter’s VO_2_max [17]. These tasks alone increase the risk of cardiovascular strain, and the addition of PPE multiplies this risk by elevating heart rate, increasing myocardial oxygen demands, and contributing to the heightened risks of sudden cardiac events that are observed in firefighters. Furthermore, PPE blocks evaporative and convective colling, which forces blood to the skin, thus further increasing cardiovascular strain and accelerating fatigue onset [6,9]. Together, these thermoregulatory, metabolic, and cardiovascular burdens clearly demonstrate that PPE crucially changes the physiological environment in which firefighters must work. To ensure individual, crew, and community safety, firefighters must perform their duties at the highest level possible. However, a decrease in the core temperature gradient may diminish performance [8,18]. Although elevations in core temperature can be critical, they are often proceeded by increases in skin temperature, because skin temperature is more strongly influenced by environmental factors [19]. In fact, elevations in skin temperature have been shown to decrease aerobic performance even when paired with stable core temperatures [8]. Even when core temperature increased, skin temperature was reduced and performance was maintained; conversely, when core temperature was kept constant and skin temperature was elevated, time to exhaustion and aerobic capacity greatly decreased [8,18]. When the core–skin temperature gradient narrows, aerobic capacity and thus VO_2_max decrease, leading to shorter times to exertion and increased fatigue perception [8,20]. Elevated skin temperatures reduce VO_2_max because they increase cutaneous blood flow, which pulls blood from the working muscles and brain [9]. This redistribution reduces maximal cardiac output, cardiac filling, and ultimately oxygen distribution [10].

It is well established that in isolation both PPE and decreases in the core–skin temperature gradient increase cardiovascular strain and hinder performance [8,9,12,18,20]. However, little research has directly examined the effects on PPE on the core–skin temperature gradient in recruit firefighters. Because this gradient represents a critical aspect of heat transfer from the core to the environment, gradient reductions may increase heat storage, limit heat dissipation, and decrease aerobic performance [8,18,20]. Given that firefighting requires sustained high intensity physical effort, even small impairments in thermoregulation and performance can have detrimental effects. Therefore, the purpose of this study was to examine the immediate effects of donning PPE on the core–skin temperature gradient. Because core temperature is influenced by exercise intensity and skin temperature by environmental conditions, timepoints were chosen at the end of recovery periods where PPE was off and then soon after PPE was put on [7,10]. These timepoints will allow for the minimization of confounding variables such as exercise intensity and environmental conditions. Additionally, because very few studies have examined the immediate effects of donning PPE, as opposed to longer-term PPE exposure, this study will help establish more accurate baseline measures that reflect the thermoregulatory impairments associated with the immediate addition of PPE.

## 2. Materials and Methods

### 2.1. Study Design

An observational, repeated-measures study design was used to examine the effect of firefighter PPE on the relationship between skin temperature and core temperature during firefighting training. Data was continuously collected from a recruit cohort throughout a shift occurring during the last week of a 12-week fire academy in Georgia. This data was split into periods defined as PPE ON and PPE OFF.

The analyses for this study used data gathered during passive recovery or light activity (e.g., standing, walking, or getting ready for subsequent tasks) to minimize variability in metabolic heat production and strengthen control of potential confounding variables. Examining closely matched two-minute average values taken just before (PPE OFF) and soon after (PPE ON) donning PPE, under almost identical ambient conditions and without significant changes in physical activity, further reinforced the control conditions. This repeated-measures, within-participant design made it possible to attribute changes in skin temperature and the core–skin temperature gradient to PPE donning rather than variations in activity level or exposure to the environment.

All procedures were approved by the institutional review board (IRBnet # 2047457), and informed consent was obtained from all participants prior to participation.

### 2.2. Participants

A total of nine male firefighter recruits from a 2024 recruit class attending fire academy were included in the analysis. Inclusion criteria included being at least 18 years old and employed by a specific fire department in Georgia. Participant characteristics (mean ± standard deviation) included age, 26.4 ± 8.2 years; weight, 84.6 ± 16.7 kg; height, 177.8 ± 7.8 cm; and BMI 26.5 ± 3.3 kg/m^2^. All nine participants wore a monitor around their chest for continuous measures of skin temperature and ingested a pill for continuous measures of core temperature.

### 2.3. Instrumentation

#### 2.3.1. Skin Temperature Monitoring

Recruit skin temperature was measured using a wearable monitoring system (Calera Research, greenTEG AG, Rümlang, Switzerland). In the morning prior to testing, the skin temperature monitor was secured around the chest with an elastic strap and positioned at the midline of the chest. The temperature sensor maintained direct contact with the skin over the center of the sternum which provided a consistent skin temperature measurement site. Skin temperature was recorded at 1 min intervals and was stored in the Calera Research Tool software via Bluetooth.

#### 2.3.2. Core Temperature Monitoring

Core temperature was assessed using an ingestible electronic capsule providing continuous core temperature monitoring in the gastrointestinal tract (eCelsius Performance, BodyCAP, Hérouville-Saint-Clair, France). Participants ingested the pill immediately upon waking before coming in for testing which provided enough time for it to reach the gastrointestinal tract while reducing the possibility of early excretion prior to testing. Core temperature was transmitted from the capsule to the eViewer Performance monitor. Recorded data was then downloaded from the monitor via USB connection in 30 s intervals. Exclusion criteria for ingesting the electronic capsule included any of the following: body weight below 36.5 kg (80.5 lb), obstructive gastrointestinal disease, history of gastrointestinal surgery, implanted medical device, pregnant, swallowing disorder, and/or had a scheduled MRI scan during the experimental period. The exclusionary criteria checklist was provided immediately before providing the electronic capsule to each participant. Ingestible telemetry pills have been shown to be valid for monitoring core temperature during periods of exercise and heat stress [21].

### 2.4. Experimental Conditions

**Figure 1:**
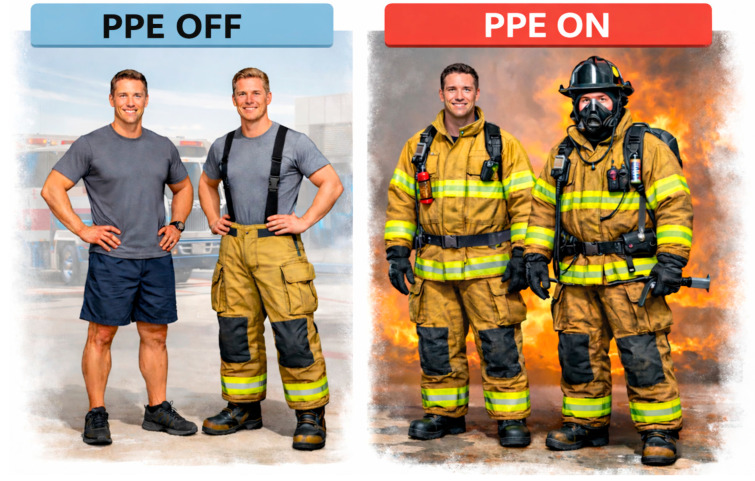
Minimum and maximum amount of clothing worn for the PPE OFF and ON conditions. Note: This figure was created using AI-generated imagery.

Time periods of PPE ON and PPE OFF activity were recorded by the investigator for each participant during the monitoring of the recruits. PPE ON was defined as wearing full structural firefighter protective gear, including turnout gear and associated insulating layers. The minimum requirement for being categorized as wearing PPE includes wearing the insulated jacket, pants, gloves, and boots. The helmet and SCBA were not required to be categorized as PPE ON due to the helmet coming on and off frequently. PPE OFF periods included times when participants were not wearing full PPE which includes no helmet, jacket or gloves. The pants and boots were allowed during PPE OFF periods. Figure 1 provides examples of the minimum and maximum amount of clothing worn in the PPE OFF and ON conditions. Participants transitioned continually between conditions as part of their firefighting training activities.

The length of time that PPE was ON and OFF was determined by the training exercise of the recruits. These periods ranged in length from 15 to 49 min for ON and from 9 to 63 min for OFF. All data were collected in the same location outdoors throughout one day from early morning until late afternoon. The weather conditions in the early morning consisted of ambient temperatures between 72 and 75° Fahrenheit (F) with relative humidity of 85–90%. The ambient temperature warmed throughout the day to 83–84° F and humidity decreased to 50–55% in the late afternoon. The day remained partly sunny with light winds and no precipitation.

### 2.5. Data Processing

**Figure 2:**
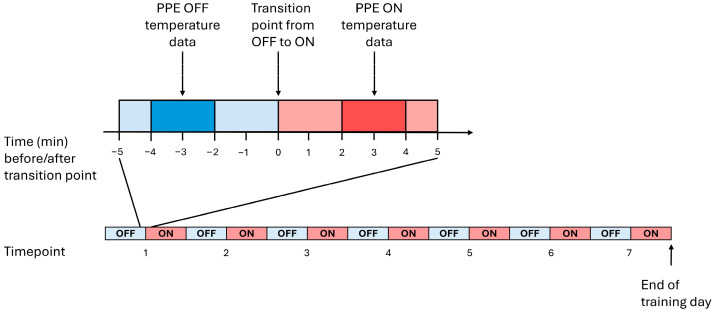
Schematic diagram depicting the time at which temperature data was collected for later analysis. Blue portions of the bar indicate that personal protective equipment (PPE) is not worn (OFF), and red portions indicate that PPE is being worn (ON). The darker blue and red areas indicate the two-minute period of data collection. The organization of the seven points at which data was collected is displayed in the lower bar.

Core temperature and skin temperature were collected continuously. Temperature data were averaged for each 30 s period by the data capture software (eViewer Performance monitor and Calera Research Tool, respectively). Throughout the day of data collection, periods of time during which PPE was worn (ON) and not being worn as recruits underwent passive recovery (OFF) were determined. The transition period that separated OFF and ON periods was identified. A two-minute period before the end of each OFF period and near the beginning of each subsequent ON period was determined These timepoints captured the core and skin temperature at the end of a recovery period (OFF) and when PPE was donned without a change in ambient temperature or physical activity (ON). The average values for core temperature, skin temperature, and the temperature gradient between (core temperature−skin temperature) them were computed for each participant at these OFF–ON pairings. There was a total of seven such pairings, or 14 total data points for analysis, and these are shown in Figure 2. Two-minute averages were used to account for physiological noise that could confound data collection.

### 2.6. Statistical Analysis

To evaluate the effect of wearing PPE on the core–skin temperature gradient, the gradient values for OFF periods were compared to their subsequent ON period for each of the seven timepoints. A factorial repeated-measures analysis of variance (RMANOVA) was used to compare the effects of PPE status (2 levels: OFF and ON), time (7 levels: timepoints 1–7), and their interaction effect on temperature gradient. Similarly, RMANOVAs were used to evaluate the effects of these variables on core temperature and skin temperature.

All analyses were completed with SPSS, version 31 (IBM, Armonk, NY, USA) using an alpha level of 0.05. Data for each participant at each timepoint were screened for outliers (z-score > 3.29 standard deviation units from the group mean) and for the assumption of normality using the Shapiro–Wilk test (α = 0.05). The RMANOVA procedure is robust to violations of normality; therefore, no transformation of the data was made to account for any such reported violations. Significant interaction effects were followed up with Bonferroni-adjusted post hoc tests as needed. Simple effects are reported but did not undergo post hoc testing if there was a significant interaction effect present. Effect size is reported as partial eta squared (ƞ^2^) for RMANOVAs and are interpreted according to established guidelines: small (ƞ^2^ = 0.01), medium (ƞ^2^ = 0.06), and large effects (ƞ^2^ = 0.14). Descriptive results are presented as: mean (standard deviation). Post hoc test results for significant interaction effects include 95% confidence intervals as well. All graphs were constructed with GraphPad Prism version 10.0.0 for Windows (GraphPad Software, Boston, MA, USA).

## 3. Results

One participant was missing core temperature data for the entire day of data collection due to device malfunction. Data from this participant was removed from analysis via listwise deletion, leaving data from eight participants for analysis.

**Figure 3:**
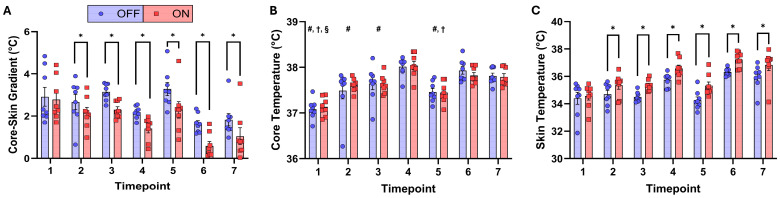
Bar graphs with individual data points for (**A**) temperature gradient, (**B**) core temperature, and (**C**) skin temperature data. Average and individual values for the participants with PPE off (OFF; blue bars and dots) and on (ON; red bars and squares) at each timepoint. Bars represent mean values for the indicated condition and timepoint. Whiskers represent the standard error of the mean value. Note: Interaction effects are signified by: * = significant difference between conditions at a given timepoint; main effects of time are signified by: # = significantly different than timepoint 4; † = Significantly different than timepoint 6; § = significantly different than timepoint 7.

A written summary of the RMANOVA results is provided in the text below. The results for temperature gradient, core temperature, and skin temperature are displayed visually in Figure 3. Accompanying tables provide complete RMANOVA results (Table 1), and relevant post hoc test results (Table 2, Table 3 and Table 4).

### 3.1. Temperature Gradient

There was a significant interaction effect between PPE status and time on temperature gradient (*p* < 0.001), which was higher in OFF than in ON for timepoints 2, 3, 4, 5, 6, and 7. Results are presented visually in Figure 3A and complete Bonferroni-adjusted post hoc test results are presented in Table 2. There were also significant effects of PPE status (*p* < 0.001) and time (*p* = 0.003) on temperature gradient, but these results are not interpreted further due to a significant interaction effect.

### 3.2. Core Temperature

There was no interaction effect (*p* = 0.445) or main effect of PPE status (*p* = 0.906) on core temperature. There was a main effect of time on core temperature (*p* < 0.001), which was highest at timepoints 4, 6, and 7 compared to earlier timepoints. Results are presented visually in Figure 3B and complete Bonferroni-adjusted post hoc test results are presented in Table 3.

### 3.3. Skin Temperature

There was a significant interaction effect between PPE status and time on skin temperature (*p* < 0.001), which was higher in OFF than in ON for timepoints 2, 3, 4, 5, 6, and 7. Results are presented visually in Figure 3C and complete Bonferroni-adjusted post hoc test results are presented in Table 4. There were also significant effects of PPE status (*p* < 0.001) and time (*p* < 0.001) on skin temperature, but these results are not interpreted further due to a significant interaction effect.

## 4. Discussion

Firefighters are required to wear PPE for many of their occupational tasks, which may lead, in part, to an elevated risk for thermal and cardiovascular strain, as insulated PPE inhibits both evaporative cooling and convective heat loss [19,22,23,24]. The relationship between core and skin temperature while wearing PPE should be examined to help prevent impairments in firefighter health, safety, and performance [25,26,27]. Thus, the physiological sequence examined in this study begins when PPE is donned, which immediately alters the skin’s surface temperature [28]. By trapping heat and humidity, structural firefighting PPE creates an enclosed, low-airflow microclimate that quickly raises skin temperature [29,30]. The efficiency of conductive heat transfer from the body’s core to the skin decreases as skin temperature rises toward core temperature and the core–skin temperature gradient narrows [31]. This decrease in the gradient indicates a significant disturbance of thermoregulatory equilibrium that may take place prior to detectable increases in core temperature [32]. In order to isolate the acute thermal effects of PPE donning in live training conditions, this study concentrated on the immediate transition from PPE OFF to PPE ON during periods of light activity.

The main finding of this study was that among firefighter recruits, the core–skin temperature gradient significantly decreased when recruits put on full PPE. Specifically, the narrowing of the temperature gradient was driven by an increase in skin temperature as opposed to a change in core temperature. On average, the gradient dropped by about 0.72 °C after putting on PPE. Large effect sizes appeared for both the gradient and skin temperature responses. This shows that wearing PPE explained a substantial portion of the physiological variation within the immediate transition window. Core temperature did not significantly change which is a notable finding as it suggests that changes in the core–skin temperature gradient, detectable from skin monitors, may be a precursor to increases in core temperature and thus heat-related illnesses. This is consistent with previous studies that show core temperature rapidly rises when there is an increase in skin temperature that meets or exceeds core temperature [25,27]. These results indicate that PPE may reduce the body’s ability to release heat from the skin to the external environment, therefore altering the heat production and dissipation relationship within minutes of donning PPE [19,23,24].

Heat transfer from the core to the environment requires both a temperature gradient and skin environment interaction. For heat dissipation to occur, increases in skin temperature should be accompanied by the stabilization or decreasing of core temperature. However, PPE interferes with the skin to environment relationship by trapping heat and limiting airflow, thereby slowing or attenuating the heat transfer process [19,22,23]. Structural turnout gear creates a hot and humid environment at the skin surface. This reduces airflow and limits sweat evaporation, especially when local humidity gets close to saturation and the vapor pressure difference decreases [22,23]. The transition period in this study was short, but even during this short observation window, the rapid rise in skin temperature shows that heat dissipation pathways are altered within two minutes of putting on PPE [24,33]. As skin temperature rises closer to core temperature, conductive heat transfer decreases. This reduces heat flow from the core and raises the risk of internal heat buildup if the PPE remains on during prolonged firefighting operations [34].

The increase in body temperature from donning PPE may place immediate strain on the cardiovascular system as blood shifts toward the skin to increase heat dissipation. This reallocation of blood to the skin as well as the demands of working muscle may push the heart to work harder to maintain adequate blood pressure and cardiac output which has been shown in live-fire situations [24,35,36]. This strain on the cardiovascular system may occur before detecting a substantial rise in core temperature. It is important to assess these immediate thermal and cardiovascular changes as they may influence firefighting performance and safety. The increased physiological strain can lead to fatigue, impaired decision-making, and reduced physical working capacity which may be detrimental to the overall safety of the firefighter as well as those involved in the emergency [37,38].

To better understand how this affects firefighters in the field, future explorations should examine how decreases in the core to skin temperature gradient affect firefighter performance and health during the initial stages as well as throughout extended firefighting activities. Determining how these immediate gradient changes influence the functional ability of firefighters will help fire departments determine their significance and adjust safety thresholds as needed. Future studies should also investigate whether a narrowed temperature gradient leads to unmanageable heat stress or reduced performance during longer response situations following this early physiological shift.

One limitation of this study was that the type and intensity of work performed during training were not standardized, as observations were made during a typical fire academy training day to prioritize field generalizability rather than experimental control. Although the analyses focused on closely timed PPE OFF–ON transitions during periods of light activity, it is possible that heat accumulated in the body from prior exertional activities which may have influenced baseline temperatures. In addition, ambient temperature and humidity were recorded throughout the day but not measured continuously which limited the ability to determine the environmental contributions to thermal strain; however, the short two-minute pairing windows help mitigate this concern by minimizing environmental and activity variability. Finally, the sample size was small and limited to firefighter recruits which may reduce generalizability. However, the repeated-measures design and multiple OFF–ON comparisons within each participant provided increased confidence that the observed narrowing of the core–skin temperature gradient was attributable to PPE donning.

This study demonstrates that thermal strain begins as soon as PPE is donned. The gradient between core and skin temperature narrows within minutes due to immediate increases in skin temperature with suggests that physiological processes that contribute to hyperthermia start before the strenuous work of firefighting begins. Understanding that these issues start early may help reinforce the importance of implementing proper hydration regiments, cooling strategies, and PPE off recovery periods as a way to slow the rate of heat accumulation thus reducing cardiovascular strain and improving overall operational safety. This study provides actionable information for fire departments to consider when developing heat management and safety protocols during training and live-fire situations.

## 5. Conclusions

This study demonstrated that donning full PPE immediately and substantially reduced the core–skin temperature gradient, primarily by increasing skin temperature. Thus, these findings show that alterations in heat dissipation occur within minutes of putting on PPE, even during light activity. Although core temperature did not rise immediately, the rapid decrease in the core–skin temperature gradient should be considered an early indicator of thermal strain.

## Figures and Tables

**Table 1:** Inferential statistical results for all repeated-measures ANOVAs.

Dependent Variable	Effect	df	F	*p*	ƞ^2^
Temperature gradient	PPE	1, 7	66.183	<0.001	0.904
	Time	2.48, 17.37	7.503	0.003	0.517
	PPE × Time	6, 42	5.518	<0.001	0.441
Core Temperature	PPE	1, 7	0.015	0.906	0.002
	Time	6, 42	18.011	<0.001	0.720
	PPE × Time	1.84, 12.91	0.840	0.546	0.107
Skin Temperature	PPE	1, 7	90.158	<0.001	0.928
	Time	2.21, 15.47	12.224	<0.001	0.636
	PPE × Time	6, 42	4.802	<0.001	0.407

Note. PPE refers to the effect of wearing (ON or OFF) personal protective equipment; Time refers to the timepoint at which the temperature data was collected (1–7); degrees of freedom (df) used for the analysis are adjusted with Mauchleys test of Sphericity, where applicable; partial eta squared (ƞ^2^) is a measure of effect size interpreted as follows: small (ƞ^2^ = 0.01), medium (ƞ^2^ = 0.06), and large (ƞ^2^ = 0.14) effects.

**Table 2:** Bonferroni-adjusted post hoc test results for differences in temperature gradient at matched timepoints, and overall difference between conditions (main effect of PPE status).

Timepoint	Mean Difference (Standard Deviation)	*p*	CI 95%
1	0.136 (0.407) °C	0.375	−0.204, 0.477 °C
2	0.513 (0.464) °C	0.017 *	0.126, 0.901 °C
3	0.839 (0.407) °C	<0.001 *	0.498, 1.181 °C
4	0.774 (0.492) °C	0.003 *	0.364, 1.184 °C
5	0.992 (0.387) °C	<0.001 *	0.668, 1.316 °C
6	1.042 (0.393) °C	<0.001 *	0.712, 1.371 °C
7	0.773 (0.438) °C	0.002 *	0.470, 1.139 °C
Overall	0.724 (0.252) °C	<0.001 *	0.514, 0.935 °C

Note. Mean difference is calculated as the OFF value minus the ON value; * = significant difference.

**Table 3:** Bonferroni-adjusted post hoc test results for differences in core temperature at different timepoints.

Timepoint	1	2	3	4	5	6	7
**1**	-	-	-	-	-	-	-
**2**	−0.438 (0.345)	-	-	-	-	-	-
**3**	−0.526 (0.320)	−0.088 (0.223)	-	-	-	-	-
**4**	−0.917 (0.271) *	−0.479 (0.150) *	−0.391 (0.127) *	-	-	-	-
**5**	−0.341 (0.356)	0.096 (0.382)	0.184 (0.416)	0.575 (0.339) *		-	-
**6**	−0.768 (0.300) *	−0.33 (0.221)	−0.242 (0.235)	0.149 (0.167)	−0.427 (0.223) *		-
**7**	−0.689 (0.260) *	−0.251 (0.320)	−0.163 (0.371)	0.228 (0.289)	−0.348 (0.243)	0.079 (0.246)	-

Note. Displayed mean differences along with (standard deviations) are calculated as the core temperature in the column timepoint minus that of the row timepoint; * = significant difference.

**Table 4:** Bonferroni-adjusted post hoc test results for differences in skin temperature at matched timepoints, and overall difference between conditions (main effect of PPE status).

Timepoint	Mean Difference (Standard Deviation)	*p*	CI 95%
1	−0.169 (0.425) °C	0.297	−0.525, 0.186 °C
2	−0.655 (0.338) °C	0.001 *	−0.938, −0.372 °C
3	−0.863 (0.388) °C	<0.001 *	−1.187, −0.539 °C
4	−0.824 (0.560) °C	0.004 *	−1.293, −0.356 °C
5	−1.067 (0.320) °C	<0.001 *	−1.335, −0.800 °C
6	−0.912 (0.359) °C	<0.001 *	−1.213, −0.612 °C
7	−0.871 (0.462) °C	0.001 *	−1.257, −0.486 °C
Overall	−0.766 (0.229) °C	<0.001 *	−0.957, −0.575 °C

Note. Mean difference is calculated as the OFF value minus the ON value; * = significant difference.

## Data Availability

The original data presented in the study are openly available through Open Science Framework at: https://osf.io/84fru/overview?view_only=b2e4f5bfb41d4cc5bdbdcf3b11d2c48f. Last updated 5 March 2026.
